# FIGO position statement on postpartum intrauterine devices (PPIUD)

**DOI:** 10.1002/ijgo.70146

**Published:** 2025-04-26

**Authors:** Anita Makins, Azra Ahsan, Marium Waqas, Asha Kasliwal, FIGO Committee on Contraception, Asha Kasliwal, Azra Ahsan, Aparna Sridhar, Cuauhtemoc C. González, Wai‐cheung Lam, Silvia Bonsergent, Felix Momat, Helena K. Kallner, Sara Ooi, Nomi Fuchs‐Montgomery, Anita Makins, Monica Kerrigan, Ian Askew

**Affiliations:** ^1^ Nuffield Department of Women's and Reproductive Health University of Oxford Oxford UK; ^2^ FIGO Committee on Contraception London UK; ^3^ Association for Mothers and Newborns (AMAN) Karachi Pakistan; ^4^ National Committee for Maternal and Neonatal Health (NCMNH) Karachi Pakistan; ^5^ Manchester University NHS Foundation Trust Manchester UK

**Keywords:** contraceptive counseling, healthcare integration, immediate postpartum contraception, long‐acting reversible contraceptives (LARCs), postpartum family planning (PPFP), postpartum intrauterine device (PPIUD), task sharing, unmet contraceptive need

## Abstract

Postpartum contraception is a critical intervention to address the unmet need for family planning, which affects over 218 million women globally, predominantly in low‐ and middle‐income countries (LMICs). Immediate postpartum family planning (PPFP) offers a unique opportunity to provide contraception during a crucial health‐seeking encounter, particularly given the rise in institutional births worldwide. Short inter‐pregnancy intervals are associated with increased maternal and neonatal morbidity and mortality, making timely postpartum contraception essential. The postpartum intrauterine device (PPIUD), a long‐acting reversible contraceptive (LARC), is a highly cost‐effective, non‐hormonal method with a low failure rate. It can be safely inserted immediately after vaginal or cesarean delivery, offering women a reliable and accessible option. However, successful implementation of PPIUD services requires overcoming barriers such as fragmented health services, provider bias, sociocultural misconceptions, and supply chain challenges. FIGO advocates for integrating PPFP, including PPIUD, into routine maternity care and emphasizes task sharing, community engagement, and comprehensive counseling as critical strategies. By incorporating postpartum contraception into standard maternal care, particularly in LMICs, health systems can improve maternal and child health outcomes, advance Sustainable Development Goals (SDGs), and empower women to make informed reproductive choices.

## 1 THE IMPORTANCE OF POSTPARTUM CONTRACEPTION

It is estimated that there are more than 218 million women with an unmet need for contraception and that the vast majority are living in low‐ and middle‐income countries (LMICs).^1^ WHO defines the unmet need for family planning as “those women who are fecund and sexually active but are not using any method of contraception, and report not wanting any more children or wanting to delay the next child”.^2^, ^3^ In many parts of the world, access to health is limited. Therefore, it is imperative that the benefit of each health‐seeking encounter is maximized through integrated services. Traditionally, contraception has been offered to women when they attend their 6‐week postnatal check, a universally implemented follow‐up for women after birth. Similarly, many women were told to go to their local family planning or sexual health clinic. In reality, this extra step is frequently hard to achieve as women are busy with their families and newborn, tending to neglect their own health. Unsurprisingly, there is therefore a documented high unmet need for contraception during the postpartum period.^4^

Globally, the percentage of births attended by skilled health personnel increased from 64% in 2001–2007 to 84% in 2015–2021. In sub‐Saharan Africa, over the same period, the indicator rose from 43% to 64%. In other regions, namely Europe, North America, and Central Asia, near‐universal or universal coverage was recorded over the period of interest.^5^ The fact that institutional birth rates have increased globally means there is now an invaluable opportunity to provide contraception to women before they leave the maternity unit. This is particularly valuable in countries where access to health care is limited.

Short inter‐pregnancy intervals exacerbate maternal morbidity and mortality and result in worse neonatal and childhood outcomes. Birth spacing of less than 24 months has been associated with increased rates of miscarriage, planned abortion, maternal mortality, pre‐term labor, small for gestational age infants, and increases in malnutrition and mortality in children under the age of 5 years.^6^, ^7^ Reducing the unmet need for contraception is one of the 17 Sustainable Development Goals (SDGs)—target 3.7, and advances would also impact targets 3.1 (maternal mortality) and 3.2 (reduce deaths of newborns and under‐5 s).^8^ Contraception alone is postulated to have the potential to reduce maternal mortality by 30%.^9^

Contraceptive choices for postpartum women have been largely restricted to temporary methods. This proves to be problematic when supply chains are broken, as was the case during the COVID‐19 pandemic. With the threat of unstable funding for contraceptive methods in LMICs, as well as the possibility of new pandemics affecting international trade and the continuous demand for contraception, there is a need to refocus and motivate eligible couples for LARCs such as the PPIUD, with significantly lower failure rates.^10^, ^11^

Therefore, counseling on immediate postpartum family planning (PPFP) and subsequent access to LARCs, such as the PPIUD, offer women the opportunity to take a more reliable break from childbearing than short‐acting methods. This allows them to look after their own health and that of their children, as well as consider further education and/or supporting the family financially if they choose to. This in turn would help to break the cycle of poverty.

## 2 THE POSTPARTUM INTRAUTERINE DEVICE (PPIUD)

The PPIUD is a LARC that contains no hormones and is extremely cost‐effective. It has been available internationally for many years, with many doctors, nurses, and midwives around the world being comfortable with inserting and removing the device. However, experience with insertion in the immediate postpartum period is less widespread and globalized. Its insertion into the postpartum uterus by hand was first described in the 1960s,^12^ but the method went out of favor as very high expulsion rates were reported. In the recent past, three large studies have examined insertion using the Kelly forceps methodology, reporting expulsion rates similar to those observed with interval insertion of the method. Jhpiego launched a multi‐country initiative between 2010 and 2013,^13^ as did FIGO between 2013 and 2020^14^, ^15^ and between 2012 and 2022 in 52 health facilities across Pakistan.^16^

## 3 THE METHODOLOGY BEHIND PROVISION OF PPIUD

After in‐depth non‐coercive counseling, a woman who chooses the PPIUD method can have it inserted immediately after birth, once the infant and placenta are delivered. This is often described as a “post‐placental” insertion and allows for a “one‐stop” approach, negating the need for any repeat examination or postnatal visit. For those women who decide to take up the method after birth, or if the maternity unit is unable to provide the service immediately at birth, the PPIUD can also be inserted any other time up until 48 h after delivery, before discharge from the health facility. For vaginal deliveries, the insertion technique uses the 32‐cm long curved Kelly placental forceps. This technique has been well described in the literature^17^, ^18^, ^19^, ^20^, ^21^, ^22^ and ensures a high fundal placement when compared to using the inserter's hand or other instruments, such as the Rampley sponge holding forceps, which is much shorter. Insertion after 48 h is not recommended owing to the known higher risk of complications.^23^

If a woman is having a cesarean delivery, the PPIUD can also be inserted through the surgical incision to the uterus once the infant and placenta have been removed and any bleeding is under control. The method can involve removing the intrauterine contraceptive device from the inserter and introducing it in the fundus of the uterus by hand, or it can be introduced using the inserter. Either method is acceptable as long as it is positioned at the fundus of the uterus and lies longitudinally, with the threads in the cavity and stretched out towards the internal cervical os. While closing the uterine incision, it is important to ensure that the IUD thread is not entrapped in the sutures.

FIGO has published guidance on the insertion of PPIUDs after both vaginal delivery and intra‐cesarean, including videos on the FIGO website demonstrating the technique: https://www.figo.org/what‐we‐do/figo‐projects/ppiud‐project/ppfp‐counselling‐and‐ppiud‐insertion‐training‐videos.

## 4 BARRIERS AND SOLUTIONS TO IMPLEMENTATION OF PPIUD SERVICES

### 4.1 Fragmented services

Setting up a PPFP and PPIUD service can be difficult in some contexts because they tend to span a variety of departments, including maternity services, sexual health, and primary care. Coordinating action between these departments, particularly in high‐income country settings where services become super specialized, can be difficult. Coordination and agreement among all stakeholders from the outset are key to ensuring a functional service.^24^, ^25^

### 4.2 The importance of task sharing

Insertion of the PPIUD is a task that has been traditionally performed by doctors. Limiting its postpartum insertion to doctors only will limit its accessibility to those women who have a normal birth and never see a doctor during their care. These are the majority of women. Task sharing is therefore an invaluable tool to increase access to postpartum contraception and PPIUD services. Many studies document huge increases in the accessibility of PPIUD when task sharing to nurses and midwives occurs in both Africa and Asia.^14^, ^26^, ^27^ Task sharing has also been found to be safe with no concerns regarding an increase in complications when insertions are performed by properly trained non‐medical staff.^14^

### 4.3 Community engagement

Community engagement is key to ensuring a successful program. In many parts of the world, there are deeply engrained misconceptions, myths, and misunderstandings regarding postpartum contraception and the IUD in particular. This can come both from the women and the providers. It is imperative that from the outset the local community is engaged in the service and that providers of all levels are trained in counseling, enabling them to explain the pros and cons of the method and dispel any myths.^28^, ^29^, ^30^

## 5 FIGO RECOMMENDATIONS

1. **Postpartum contraception services should be provided in maternity services as part of continuity of care for the woman and to improve maternal and reproductive health outcomes.**
  Healthcare providers should be encouraged to integrate PPFP into their practice and ensure their teams are trained in providing these services. Prioritizing PPFP as part of standard postpartum care, rather than referring patients to exclusive family planning clinics, is essential for optimizing access to postpartum contraceptive options. Immediate PPFP is crucial to meet unmet needs and unintended pregnancies, and reduce health risks associated with closely spaced pregnancies.
  There is a move to integrate family planning services into Emergency Obstetric and Newborn Care (EmONC). This would ensure that EmONC services offer contraception as part of routine maternity care.
2. **When commencing postpartum contraception services, it is imperative that myths and misconceptions are addressed on both the provider's and the woman's side.**
  Provider‐related bias and sociocultural beliefs of the woman and her family impact hugely on the uptake of PPIUD. Studies have highlighted barriers such as insufficient knowledge, myths, religious beliefs, provider biases, preference for short‐acting methods, spousal pressure, and fear of side effects and complications. Furthermore, healthcare providers' personal beliefs, shaped by cultural or religious values or inadequate training, may hinder comprehensive PPFP services. It is recommended that specific targeted interventions involving the community and healthcare providers should be developed alongside the provision of services.
3. **Counseling on contraception should be offered throughout the antepartum, early intrapartum, and immediate postpartum periods, with balanced, coercion‐free discussions over multiple encounters to ensure women have the time and information needed to make an informed choice.**
  This approach allows women to consult with her family and make decisions before labor begins. For those arriving in labor without prior antenatal care, early labor presents an opportunity to counsel the woman and her family members, especially male partners who are often present and may be involved in decision‐making.
  Contraception counseling should also be offered immediately postpartum for those women who report late in labor, when women may be more receptive to the discussion. To facilitate this, counseling in antenatal clinics as well as round‐the‐clock counseling should be available in delivery rooms, with additional family planning counselors hired if necessary.
  However, regular supervision and monitoring of the counselors is required to sustain quality.
  In high‐volume settings, pre‐recorded videos can serve as effective counseling tools when individualized sessions are not feasible. These can be played in waiting rooms and corridors. Similarly, leaflets containing key information on each method can be useful.
4. **Maternity services should offer PPIUD as a key option among the array of PPFP methods. PPIUD is highly cost‐effective, long‐acting (remaining effective for 10–12 years), reversible, has a very low failure rate, and is non‐hormonal (no interference with breastfeeding).**
  The hormone‐based levonorgestrel‐releasing intrauterine system can also be offered during the postpartum period. It falls in Medical Eligibility Criteria for Contraceptive Use (MEC) category 2 for breastfeeding women, remains effective for 8 years, and may be particularly suitable for women with a history of heavy periods or anemia. A key barrier to accessing implants and intrauterine systems in LMICs is the high cost driven by patent laws, making these effective contraceptive methods unaffordable for many. To address this inequity in access to modern contraception, efforts should focus on making these options affordable and accessible to women in LMICs who need them most.
5. **Counseling specific to PPIUD needs to provide comprehensive information on the device's efficacy, benefits, potential side effects, and complications.**
  Counseling should include detailed discussions on common concerns, such as menstrual irregularities, missing threads, signs of expulsion, and pain. Since PPIUD threads are not trimmed at insertion, women should be advised that threads may protrude as the uterus contracts but that this is incredibly rare. Were it to occur, they should be instructed to gently push the threads back into the vagina and report to their provider. Clinicians should be aware that because bleeding and discomfort are common postpartum and after IUD insertion, acceptance of the device may be higher immediately after childbirth than with interval insertion.
6. **If the PPIUD is chosen, it should be administered at or immediately after birth (within 48 h) and before hospital discharge.**
  If a PPIUD is not inserted within 48 h of delivery, it should be delayed until 4–6 weeks postpartum. If women are unable to receive their preferred contraceptive method before discharge from maternity services, they should be offered effective bridging contraception to ensure continuous protection.
7. **A PPIUD can be safely inserted during cesarean delivery, immediately after the infant and the placenta have been delivered. Care should be taken to place the PPIUD at the fundus, to straighten the threads towards the cervical os, and not to inadvertently include the threads in the closure of the uterine incision.**
  With 21.1% of women worldwide having a cesarean delivery—ranging from 5% in sub‐Saharan Africa to 42.8% in Latin America and the Caribbean—the option to insert a PPIUD during cesarean delivery is an effective strategy.^31^ The PPIUD can be placed immediately after delivering the infant and placenta and before closing the uterus, with studies showing comparable effectiveness and expulsion rates to insertion after vaginal delivery. After insertion, care should be taken to not inadvertently include the IUD threads into closure of the incision.
  Given the high rates of emergency cesarean deliveries, PPIUD insertion should also be integrated into EmONC services. If the woman has consented in advance and there are no contraindications, such as prolonged rupture of membranes or evidence of chorioamnionitis, this is deemed safe.
8. **A PPIUD can be safely inserted after vaginal delivery using either long 33‐cm Kelly forceps or a purpose‐built device.**
  Proper placement high in the uterine cavity at the fundus is essential to minimize expulsion. When correctly positioned, evidence suggests that expulsion rates are comparable to those of interval insertions (3%). It is not recommended to insert the device using the 24‐cm tissue or sponge forceps as it is not long enough and does not reach the uterine fundus, increasing the risk of expulsion.
  It has been noted that there is a better chance of thread visibility after insertion using the purpose‐built device. However, given the longer threads, a follow up visit is required at 2 weeks to trim these threads. The purpose‐built device is also prohibitively expensive for many countries providing the service free at the point of care.
9. **PPIUD insertion is contraindicated in the presence of infection (chorioamnionitis) or prolonged rupture of membranes (>18 h) due to the risk of infection.**
  In addition, if there is a history of prolonged labor, particularly when managed by traditional birth attendants, insertion of a PPIUD is not advised given that the risk of infection is high. In the case of an ongoing postpartum hemorrhage (PPH), this should be dealt with first and the IUD inserted only once the PPH is under control and mitigating action has been taken successfully.
  Clinicians should also bear in mind the standard contraindications to insertion of the copper coil at any time, including any allergy to copper, Wilson's disease, a grossly distorted uterine cavity, malignant trophoblastic disease, pelvic tuberculosis, and cancers of the genital tract (cervical or endometrial).
10. **PPIUD insertions are associated with very low complication rates, with minimal risk of infection and very few cases of perforation reported.**
  However, misperceptions among healthcare providers and patients can lead to unnecessary removal due to mistaken concerns of infection. In a large study of 146 318 PPIUD insertions in Pakistan, only two cases of uterine perforation were reported. There were none reported in the FIGO series of 37 383 across six countries.
  Nevertheless, comprehensive training of providers and appropriate patient selection are essential to ensure safe and effective PPIUD insertion. To minimize the risk of perforation, it is recommended that healthcare providers carefully trace the entire cervix before insertion to avoid accidental perforation, especially of the posterior vaginal fornix.
11. **Routine post‐insertion check‐ups are not required for IUDs in a healthcare facility.**
  For IUDs placed in the first 48 h postpartum, it is recommended that the woman self‐checks for the threads per vagina at 4–6 weeks. If they are not present, she should use another form of bridging contraception and go and see a clinician for further investigation.
  Although self‐checking is sufficient, many units routinely organize a follow‐up with a clinician 4–6 weeks after insertion to check that the threads are visible and that the IUD is in situ.
  For IUDs, especially with longer threads, women can be advised to attend an earlier follow‐up and not to pull on any threads that may become visible outside the vagina. Instead, they should consult their provider for trimming.
12. **Missing strings or lost threads can occur in up to 30% of PPIUD cases and are more common in women who had it inserted during a cesarean delivery.**
  This rate often decreases once menstruation resumes. Missing threads are generally not a cause for concern. Patients should be reassured but advised to use bridging contraception until a transvaginal sonography (TVS) can be organized to ensure the device is still in utero. If the device is not visible on TVS, an abdominal radiograph may be necessary. For cases involving migrated devices, laparoscopy or laparotomy may be required. If the device is found in utero, retrieval of the threads is only necessary if the woman wants the device to be removed. This can be performed using a Cytobrush or plastic thread retriever and, if unsuccessful, a hysteroscopy can be considered. Metallic thread retrievers and artery forceps should only be used by senior clinicians as the risk of inadvertently perforating the uterus may be high.
13. **Task sharing is strongly recommended as an effective approach to expand access to PPIUD services.**
  With proper training and empowerment, mid‐level providers are equally skilled in delivering safe PPIUD services. This is particularly beneficial in facilities where women primarily interact with midwives during delivery. Limiting insertion to doctors only will reduce access to this highly efficacious method.
14. **To ensure the continuous availability of contraceptives, a robust supply chain system should be established, guaranteeing that contraceptives are accessible in labor rooms and operation theaters 24 h a day.**
  Currently, healthcare providers lack clarity on how to secure a sustainable supply of contraceptive commodities for their facilities. Addressing this gap should be a government priority.
15. **To sustain the provision of PPFP, including PPIUD services, both pre‐service and on‐the‐job training should be provided for all healthcare providers.**
  Nursing and medical curricula should be updated to incorporate PPFP concepts into pre‐service education, practical training, and examinations. Regular training on the labor ward using hands on mannequins of the postpartum uterus and Kelly forceps or the purpose‐built device should be developed and run cyclically so that there is continuous learning.

## 6 CONCLUDING STATEMENT

PPFP is a key intervention for the reduction of maternal and neonatal morbidity and mortality, by reducing the unmet need for contraception in the postpartum period. It is essential that appropriate balanced non‐coercive contraception counseling services are provided in the antepartum, early intrapartum, and immediate postpartum periods for all women. PPIUD is a highly cost‐effective, efficacious LARC that should be made available in maternity services to all women who choose it. The recent FIGO and ICM (International Confederation of Midwives) joint statement on contraception supports the recommendations.^32^

### 6.1 FIGO's commitments

- To advocate for access to contraception throughout a woman's reproductive years and in particular during the immediate postpartum period.
- To facilitate and provide training materials to help clinicians and ministries of health to provide access to contraception during the immediate postpartum period, regarding PPIUD in particular.

## AUTHOR CONTRIBUTIONS

AM and AA devised the concept, planned and wrote the article with strong support from MW with regards to the literature review. AK reviewed the article. AM, AA and AK are members and chair of the FIGO Committee of Contraception, and this article was written as part of their involvement with the committee.

## FUNDING INFORMATION

There was no funding source for the preparation of this paper.

## CONFLICT OF INTEREST STATEMENT

The authors declare no conflict of interest.

## MEMBERS OF THE FIGO COMMITTEE ON CONTRACEPTION, 2023–2025

Asha Kasliwal (Chair), Azra Ahsan (Vice Chair), Aparna Sridhar (Past Chair), Cuauhtemoc C. González, Wai‐cheung Lam, Silvia Bonsergent, Felix Momat, Helena K. Kallner, Sara Ooi (WATOG Representative), Nomi Fuchs‐Montgomery (Associate Member); Anita Makins (Associate Member), Monica Kerrigan (Associate Member), Ian Askew (Associate Member).

## Supporting information


Data S1.

## Data Availability

Data sharing is not applicable to this article as no new data were created or analyzed in this study.
