# Supplementary material for: FIGO position statement on postpartum intrauterine devices (PPIUD)
Source: Int J Gynaecol Obstet. 2025 Apr 26;169(3):1127–32. doi: 10.1002/ijgo.70146 (PMC12093926; doi:10.1002/ijgo.70146)
Supplement: Supplementary file 1 — Data S1. [file IJGO-169-1127-s001.docx]

**PPIUD**

**The evidence supporting FIGO’s recommendations**

| **No.** | **Studies** | **Recommendation** |
| --- | --- | --- |
|  | Increasing childbirth by SBAs means there is an opportunity to offer more than just birthing.  Globally, the percentage of births attended by skilled health personnel increased from 64% in 2001–2007 to 84% in 2015–2021. In sub-Saharan Africa, over the same period, the indicator rose from 43% to 64%. In other regions, namely Europe, North America, and Central Asia, near universal or universal coverage was recorded over the period of interest. (*United Nations Children’s Fund, World Health Organization S. Births attended by skilled health personnel (%) - Joint UNICEF/WHO database. 2022.)*  Never before has the obstetric encounter for birth been more valuable than now. It will be one of the only face‐to‐face opportunities women may have with a qualified provider during the pandemic and should allow for integrated health services to be provided, with contraception being an absolute priority. The advantages of birth spacing have been well documented, and during the pandemic avoiding a pregnancy is wise for women not only on a personal level, but also from a public health perspective in terms of not adding to overburdened systems. The range of methods offered should include precise advice on LAM to enhance its efficacy, barrier methods such as condoms, plentiful supplies of the oral progestin‐only pill (a minimum of 6 months), DMPA (preferably subcutaneous rather than intramuscular to enable self‐administration), and perhaps the most advantageous of all: LARCs. **FIGO’s Contraception and Family Planning Committee joins other health authorities to voice the urgency with which maternity units across the globe need to turn their attention to offering and providing immediate PPFP to all consenting women before discharge.** (*Makins A, Arulkumaran S; FIGO Contraception and Family Planning Committee. The negative impact of COVID-19 on contraception and sexual and reproductive health: Could immediate postpartum LARCs be the solution? Int J Gynaecol Obstet. 2020 Aug;150(2):141–143. doi: 10.1002/ijgo.13237. Epub 2020 Jun 25. PMID: 32449192; PMCID: PMC9087606.*  *<https://pmc.ncbi.nlm.nih.gov/articles/PMC9087606/>)*  As rates of institutional deliveries are increasing and maternity services are often the only HFs women come into contact with, the postpartum period presents an ideal opportunity to increase women’s access to contraceptive counseling and provide them with the choice of family planning. *(Singh S, Darroch JE. Adding It Up: Costs and Benefits of Contraception Services Estimates for 2012. 2012. Guttmacher Institute and United National Population Fund (UNFPA); <http://www.guttmacher.org/> pubs/AIU-2012-estimates.pdf Accessed May 20, 2020.+ (FIGO - Technical Brief: Implementing postpartum intrauterine device (PPIUD) services in healthcare facilities)* | Contraception services should be provided as part of continuity of care for all woman of reproductive age, including those who are immediately postpartum.  HCPs, who may prioritize other routine clinical duties over family planning, should be encouraged to integrate PPFP into their practice and ensure their teams are trained in providing these services. Prioritizing PPFP as part of standard postpartum care, rather than referring patients to exclusive FP clinics, is essential for optimizing access to postpartum contraceptive options. Immediate PPFP is crucial to meet unmet needs, and unintended pregnancies, and reduce health risks associated with closely spaced pregnancies.  There is a move to integrate FP services into EmONC. This would ensure that all EmONC services offer contraception as part of routine maternity care.  All SBAs should receive training so that they can offer PPFP, including offering LARCs, to enhance effective FP options immediately after birth. |
|  | Personal bias of HCPs and myths and misconceptions should be addressed through targeted training and initiatives engaging community leaders.  A number of reasons for non-insertion in women who had previously consented to receiving PPIUDs were provider-related or bias or sociocultural beliefs of the women or their families. (*Azra Ahsan and Aleya Ali; Acceptability, continuation and complication rate of postpartum insertion of intrauterine contraceptive device among Pakistani women; Journal of Pakistan Medical Association(Vol. 73, Issue 5); DOI: <https://doi.org/10.47391/JPMA.6804%20>; <https://jpma.org.pk/index.php/public_html/article/view/6804>*)  Consistent with these findings, other studies have also reported reasons, like lack of knowledge, myths, religious beliefs, untrained providers, inclination towards other short-acting contraceptive methods, spousal pressures, and fear of complications. *(Hauck B, Costescu D. Barriers and Misperceptions limiting widespread use of intrauterine contraception. J Obstet Gynaecol Canada. 2015; 37:606-16. doi: 10.1016/S1701-2163(15)30198-5.)+*  In addition, personal beliefs of some HCPs, based on their cultural or religious values or poor training, impact the care given to women and are often in conflict with their professional responsibilities. (*Azra Ahsan and Aleya Ali; Acceptability, continuation and complication rate of postpartum insertion of intrauterine contraceptive device among Pakistani women; Journal of Pakistan Medical Association(Vol. 73, Issue 5); DOI: <https://doi.org/10.47391/JPMA.6804%20>; <https://jpma.org.pk/index.php/public_html/article/view/6804>*)  It was found that most postpartum mothers did not take the PPIUD due to misconceptions, poor attitude, utilization of herbal medicine, negative attitude of health workers, and long waiting hours for services. *(Brender Namusoke,Sr. Ronah Orishaba St. Francis School of Health Sciences: SJ-GYN/OB [Internet]. 2024 May 5 [cited 2024 Nov. 5];1(5):11. Factors contributing to low uptake of postpartum intra uterine device in youth aged 18-35 years A Cross-Sectional study at Mukono General Hospital, Mukono district. Available from: https://obsgyn.sjpublisher.org/index.php/OBGYN/article/view/14; DOI: https://doi.org/10.51168/xh55w812)*  Awareness and uptake of IPP LARCs was low despite the good awareness and uptake of contraception in general. Reasons for the poor uptake include lack of awareness, ignorance about the availability of the service in the facility, fear of side effects, and husbands’ disapproval. This makes it pertinent that couple targeted postpartum contraception counseling be included in routine contraception counseling. *(Hannatu F Kachiro, Hajara Umaru-Sule, Afolabi K Koledade, Hajaratu Umar-Sulayman: Awareness and Uptake of Immediate Postpartum Long-Acting Reversible Contraception Among Women in Northern Nigeria: Tropical Journal of Obstetrics and Gynaecology, Vol. 42 No. 1 (2024)*  In the study area, the woman is not the sole deciding person. Her husband and in-laws play a major role in decision-making related to FP devices. Myths and fear of complications related to IUDs are the major barriers. *(Vijayashree Saligrama Rajaiah, Siddalingappa Hugar, Varsha Hoogar, Harish Bekkalele Rurdresh: Barriers for acceptance of intra uterine contraceptive devices among postnatal mothers residing in rural areas of Mandya, Karnataka: A Cross – Sectional Qualitative study: Journal of Cardiovascular Disease Research, Vol 15, Issue 01, 2024)*  Consent for contraception, specifically PPIUDs, is a culturally specific topic and generalization across countries is not possible. When planning contraceptive policy changes, it is important to have an understanding of the sociocultural factors at play. *(Makins A, Taghinejadi N, Sethi M, Machiyama K, Thapa K, Perera G, Munganyizi PS, Bhardwaj A, Arulkumaran S. Factors influencing the likelihood of acceptance of postpartum intrauterine devices across four countries: India, Nepal, Sri Lanka, and Tanzania. Int J Gynaecol Obstet. 2018 Sep;143 Suppl 1:13-19. doi: 10.1002/ijgo.12599. PMID: 30225876., <https://pubmed.ncbi.nlm.nih.gov/30225876/>)*  The study shows that multiple factors that are interlinked affected the behaviors related to uptake and continuation of PPIUD. The attitude helped in shaping intention but did not always lead to the behavioral outcome of PPIUD uptake and continuation. Subjective norms had a strong influence on both intention and behavior. Behavior control belief also had an important role in the outcome with respect to PPIUD uptake and continuation. Thus, a more layered, multidimensional, and interlinked intervention is necessary to bring positive behavior changes related to PPIUD. *(Thapa, K., Dhital, R., Rajbhandari, S. et al. Factors affecting the behavior outcomes on post-partum intrauterine contraceptive device uptake and continuation in Nepal: a qualitative study. BMC Pregnancy Childbirth 19, 148 (2019). <https://doi.org/10.1186/s12884-019-2310-y>, <https://bmcpregnancychildbirth.biomedcentral.com/articles/10.1186/s12884-019-2310-y>)*  Provider KAP could be improved further through ongoing and more in-depth training to maintain providers’ knowledge, reduce provider bias and misconceptions about PPIUD eligibility, and to ensure providers understand the importance of birth spacing. *(Stone, L., Puri, M.C., Guo, M. et al. Assessing knowledge, attitudes, and practice of health providers towards the provision of postpartum intrauterine devices in Nepal: a two-year follow-up. Reprod Health 18, 43 (2021). <https://doi.org/10.1186/s12978-021-01099-7>)* | When commencing postpartum contraception services, it is imperative that myths and misconceptions are addressed on both the provider and the woman’s side. Provider-related bias and sociocultural beliefs of the woman and her family, impact hugely on the uptake of a PPIUD. Studies have highlighted barriers such as insufficient knowledge, myths, religious beliefs, provider biases, preference for short-acting methods, spousal pressure, and fear of side-effects and complications. Furthermore, HCPs’ personal beliefs, shaped by cultural or religious values or inadequate training, may hinder comprehensive PPFP services. |
|  | Balanced coercion-free counseling on contraception should also occur in the antenatal period over multiple encounters where the pros and cons of all available methods can be discussed at length. This will give the woman an opportunity to discuss the options with her friends, relatives, and husband if she chooses to, and to make the right choice before she goes into labor. (Makins et al https://obgyn.onlinelibrary.wiley.com/doi/full/10.1002/ijgo.12599)  Information about contraception after childbirth should be offered in the antenatal period to support informed decision-making and facilitate provision of contraception by maternity services.  After childbirth, effective contraception should be discussed and offered before discharge from maternity services.  *(Guidance on the provision of contraception by maternity services after childbirth during the COVID-19 pandemic – FSRH, RCOG and RCMW 2021)*  Counseling during the maternity cycle plays a vital role in increasing the uptake of PPIUCD. *Animen S, Lake S, Mekuriaw E. Utilization of intra uterine contraceptive device and associated factors among reproductive age group of family planning users in Han health centre, Bahir Dar, north West Amhara, Ethiopia, 2018. BMC Res Notes. 2018; 11:1–6. doi: 10.1186/s13104-018-4032-z.)* + (*Azra Ahsan and Aleya Ali; Acceptability, continuation and complication rate of postpartum insertion of intrauterine contraceptive device among Pakistani women; Journal of Pakistan Medical Association(Vol. 73, Issue 5); DOI: <https://doi.org/10.47391/JPMA.6804%20>; <https://jpma.org.pk/index.php/public_html/article/view/6804>*)  The present study found that FP uptake was higher when women were counselled in early labor, when they were not distressed with labor pains and could make informed choices. While the woman is in labor, her husband and other decision makers are usually around, and can become part of the decision-making process. On the other hand, when women were counseled in antenatal clinics, the FP uptake was much lower. Therefore, with a limited number of FP counsellors, it is best to place them in shifts in labor rooms to ensure round-the-clock counseling services. (*Azra Ahsan and Aleya Ali; Acceptability, continuation and complication rate of postpartum insertion of intrauterine contraceptive device among Pakistani women; Journal of Pakistan Medical Association(Vol. 73, Issue 5); DOI: <https://doi.org/10.47391/JPMA.6804%20>; <https://jpma.org.pk/index.php/public_html/article/view/6804>*)  The postpartum period is an ideal time for a women to counsel regarding contraception because after bearing the agony of childbirth, there is much acceptance to it as well as counseling and convincing is easier. *(Somila Xess, Avinashi Kujur, Geetanjali Kanwar, Era Claudius, Shankar Marshal Toppo : A Comparison Between Postpartum Contraception Choices Among Antara, Chhaya, IUCD, Their Side Effects and Drop Out in The Rural Population Attending Tertiary Care Centre of RSDKS GMC Ambikapur. DOI: 10.47009/jamp.2024.6.5.49)*  The FCHVs’ knowledge and community-based activities on PPFP remained higher than in the pre-intervention period. However, it declined when compared to the immediate post-intervention period. We propose regular supervision and monitoring of the work of the FCHVs to sustain progress. *(Dhital R, Silwal RC, Pokhrel KN, Pokhrel, S, Tuladhar H, Bright S, et al. (2021) Evaluating the impact of female community health volunteer involvement in a postpartum family planning intervention in Nepal: A mixed-methods study at one-year post-intervention. PLoS ONE 16(10): e0258834. <https://doi.org/10.1371/journal.pone.0258834>; <https://journals.plos.org/plosone/article/authors?id=10.1371/journal.pone.0258834>)*  A pre-recorded video is an effective tool to improve knowledge, attitude, and practice of PPIUDs and may be utilized in hospitals with huge obstetrics burden. However, larger studies with sufficient power are warranted to draw definite conclusion. (*Dr. Archana Bharti,Gargi Agarwal, Isha Bansal, Richa Kansal, Janhvi Yeolekar: To study the impact of counseling through pre-made video on Knowledge, Attitude and Practice of postpartum Intrauterine contraceptive devices. DOI: 10.47009/jamp.2024.6.1.223)*  Reading and counseling material for SBAs and women on Healthy Timing and Spacing of Pregnancy, listing the PPFP options, was developed, distributed, and displayed prominently at the HFs. A video highlighting the benefits of PPFP was also shown in waiting areas of antenatal clinics. (*Azra Ahsan and Aleya Ali; Acceptability, continuation and complication rate of postpartum insertion of intrauterine contraceptive device among Pakistani women; Journal of Pakistan Medical Association(Vol. 73, Issue 5); DOI: <https://doi.org/10.47391/JPMA.6804%20>; <https://jpma.org.pk/index.php/public_html/article/view/6804>*)  The intervention was implemented by FIGO in partnership with AGOTA. Specific intervention components included: (1) information education and communication materials on PPFP, including leaflets and a video that played in the waiting room; (2) provider training on PPFP counseling and PPIUD insertion techniques; (3) provision of equipment, including Kelly forceps to insert the IUD; and (4) regular monitoring and support provided by FIGO and AGOTA. Giving women informational materials on PPIUD and counseling after admission for delivery are likely to increase the proportion of women choosing PPIUD. *(Pearson, E., Senderowicz, L., Pradhan, E. et al. Effect of a postpartum family planning intervention on postpartum intrauterine device counseling and choice: evidence from a cluster-randomized trial in Tanzania. BMC Women's Health 20, 102 (2020). https://doi.org/10.1186/s12905-020-00956-0)*  Other studies have also identified leaflets provided during ANC as important for increasing uptake of PPIUD. *(Karra M, Canning D, Foster S, Shah IH, Senanayake H, Ratnasiri UD, Pathiraja RP. Location and content of counseling and acceptance of postpartum IUD in Sri Lanka. Reprod Health. 2017. <https://doi.org/10.1186/s12978-017-0304-7>.)*  Providing visual aids to women, such as leaflets, posters, flipcharts, and videos in local languages, alongside counseling services is recommended where possible.  Research conducted on FIGO’s PPIUD Initiative showed that receiving multiple counseling sessions and, therefore, increased exposure to counseling, is the only consistent factor associated with an increased uptake of PPIUD, as opposed to other factors, such as cadre type.  During the FIGO PPIUD Initiative, the role of well-trained lay FP counselors was demonstrated to be invaluable in some countries, namely Bangladesh and India, and should be considered in settings where antenatal patient flow in facilities is very high, making quality counseling impossible if only left to doctors and MWs. Similarly, in Kenya and Nepal, Community Health Volunteers were also trained to counsel women and their partners on FP in their communities, which resulted in greater acceptability of the method.  In countries with a very high flow of antenatal patients in facilities, designated FP counselors can improve counseling in both quantity and quality and should be considered if sustainable by the government in the long run.  *(FIGO - Technical Brief: Implementing postpartum intrauterine device (PPIUD) services in healthcare facilities)*  In addition, male partner involvement in FP counseling and decisions with their partners could be a key strategy to increase both PPIUD and FP uptake in Rwanda.(*Tounkara MS, Ingabire R, Comeau DL, Karita E, Allen S, Nyombayire J, et al. (2022) A mixed-methods study of factors influencing postpartum intrauterine device uptake after family planning counseling among women in Kigali, Rwanda. PLoS ONE 17(11): e0276193. https://doi.org/10.1371/journal.pone.0276193)* | Counseling on contraception should be provided throughout the antenatal period, with balanced, coercion-free discussions over *multiple* encounters to ensure women have the time and information needed to make an informed choice.  This approach allows women to consult with family and make decisions before labor begins. For those arriving in labor without prior ANC, early labor presents an opportunity to counsel the woman and her family members, especially male partners who are often present and may be involved in decision-making.  Contraception counseling should also be offered immediately postpartum for those women who report late in labor, when women may be more receptive to having the discussion. To facilitate this, round-the-clock counseling should be available in delivery rooms, with additional FP counsellors hired if necessary.  However, regular supervision and monitoring of the counsellors is required to sustain progress.  In high-volume settings, pre-recorded videos can serve as effective counseling tools when individualized sessions are not feasible. These can be played in waiting rooms and corridors of maternity units. Similarly, leaflets containing key information on each method can also be useful. |
|  | The PPIUD should be one of the methods on offer in the immediate postpartum period because it is highly cost effective, long acting, and reversible, with extremely low failure rates of less than 1%. Women can be advised that it is non-hormonal and does not interfere with breastfeeding and will remain effective in utero for up to 10 years. Makins & Cameron Post pregnancy contraception article <https://pubmed.ncbi.nlm.nih.gov/32217053/>  Immediate postpartum IUC (within 48 hours of childbirth) is safe, effective, convenient and associated with high continuation rates. *(FSRH Guidelines Intrauterine Contraception – Amended 2023)*  (Mirena???, women with history of heavy periods can be given IUS – WHO Book 2022/ MEC Category) **[Author: Please check and amend or delete.]**  For women who are willing to accept the possibility of hormonal side effects, both the progesterone implant and the IUS have now been categorized by the WHO as MEC Category 2 for breastfeeding women, and will provide 3 and up to 6 years of contraception, respectively. These methods have the added advantage of often resulting in amenorrhea, which can be a welcome break for some women or an absolute necessity for those suffering with heavy menstrual bleeding. One major issue with these two methods is that patent laws mean that they are still prohibitively expensive for procurement by most LMICs. This denies the opportunity for many women in poorer countries not only to access this effective contraceptive method, but also to access adjuvant treatment for iron deficiency anemia, a condition that we know to be highly prevalent in this context. The issue of inequality of access remains with modern contraception, as it does with other medicines. (*Makins A, Arulkumaran S; FIGO Contraception and Family Planning Committee. The negative impact of COVID-19 on contraception and sexual and reproductive health: Could immediate postpartum LARCs be the solution? Int J Gynaecol Obstet. 2020 Aug;150(2):141-143. doi: 10.1002/ijgo.13237. Epub 2020 Jun 25. PMID: 32449192; PMCID: PMC9087606. <https://pmc.ncbi.nlm.nih.gov/articles/PMC9087606/>)*  There are fewer studies looking at the insertion of the LNG-IUS postpartum, but the practice has been widely adopted in some high-income countries. In light of a randomized controlled trial (RCT) that detected decreased breastfeeding duration in the group receiving immediate insertion of the IUS, WHO recommends MEC 2 in breastfeeding mothers and MEC 1 in non-breastfeeding mothers. *(Anita Makins, Sharon Cameron; Post pregnancy contraception; Best Practice & Research Clinical Obstetrics and Gynaecology; © 2020 Published by Elsevier Ltd)*  *WHO Family Planning – A Global handbook for Providers – 2022 <https://www.who.int/publications/i/item/9780999203705> )*  Levonorgestrel intrauterine system: The 52 mg LNG-IUS can be inserted at the time of cesarean birth or immediately after vaginal birth or at any time up to 48 hours postpartum by maternity staff trained in the technique. *(Guidance on the provision of contraception by maternity services after childbirth during the COVID-19 pandemic 2021, FSRH, RCOG, RCOM)*  **Problem:** Very long threads.  **Advice to user, and pathway:** Do not pull threads; trim threads to vaginal entrance; use condoms; contact local contraceptive provider for supply of POP; contact local SRH service to arrange review and further trimming of threads (may be deferred depending on circumstances). *(Guidance on the provision of contraception by maternity services after childbirth during the COVID-19 pandemic 2021, FSRH, RCOG, RCMW)*  *The Hormonal IUD access group:* “Members of the Hormonal IUD Access Group aligned on a strategy to expand access in the context of volunteerism and contraceptive method choice… On the demand side, investments in implementation research will be critical to understanding how best to launch and scale the method, while ensuring the sustainability of multiple quality-assured suppliers with affordable public-sector pricing will be necessary on the supply side.”  *Rademacher KH, Sripipatana T, Danna K, Sitrin D, Brunie A, Williams KM, Afolabi K, Rasoanirina F, Ramarao S, Pfitzer A, Cain D, Simon M, Menotti E, Hazelwood A, Nwala AA, Saidu Z, Chowdhury R, Taiwo A, Chidanyika A, Ndirangu G, Steiner MJ, Lepine MC, Homan R, Saad A, Vivalo J, Dorflinger LJ. What Have We Learned? Implementation of a Shared Learning Agenda and Access Strategy for the Hormonal Intrauterine Device. Glob Health Sci Pract. 2022 Oct 31;10(5):e2100789. doi: 10.9745/GHSP-D-21-00789. PMID: 36316136; PMCID: PMC9622288.* | PPIUD should be offered as a key FP option, as it is highly cost-effective, long-acting, reversible, and has a very low failure rate of less than 1%. Women should be informed that the PPIUD is non-hormonal and therefore does not interfere with breastfeeding and remains effective for up to 10–12 years.  In addition, the hormone-based LNG-IUS can also be offered during the postpartum period. It is in MEC category 2 for breastfeeding women, it remains effective for 8 years and may be particularly suitable for women with a history of heavy periods or anemia.  A key barrier to accessing implants and IUS in LMICs is the high cost driven by patent laws, making these effective contraceptive methods unaffordable for many. To address this inequity in access to modern contraception, efforts should focus on making these options affordable and accessible to women in LMICs who need them most. |
|  | Key points for counseling:  Misplaced IUD, missing thread, menstrual irregularities, and pain are all associated with PPIUDs and are important reasons for dissatisfaction. Appropriate, timely, and supportive individualized care that address knowledge gaps, societal perceptions, and healthcare system challenges would certainly help in reducing dissatisfaction due to PPIUD and thereby the removal rates. *(Radhika, A.G, Gupta, R. , Kashyap, P. and Bakshi, R. (2024) User Perspective of Misplaced PPIUCD and Factors Resulting in PPIUCD Removal: Qualitative Pilot Study. Open Journal of Epidemiology, 14, 517-532. doi: 10.4236/ojepi.2024.143037.)*  (Vaginal bleeding and after pains are normally present after childbirth and are not perceived due to the IUCD, so possibility of acceptance is better in the postpartum period compared to the interval period)  PPIUD continuation rates declined considerably after the initial 6 weeks. Counseling and follow-up services for managing complications must be strengthened, especially in the first 6 weeks of PPIUD insertion, to enhance and sustain programmatic impact. **Implications:** Our findings emphasize the need to strengthen client counseling and follow-up for management of complications, especially in the first 6 weeks of insertion of PPIUDs. Ongoing programs need to address comprehensive capacity-building efforts in this regard. *(Srivastava A, Sharma S, Lalchandani K, Mohanty N, Bhatt DC, Usmanova G, et al. (2024) One-year continuation of postpartum intrauterine contraceptive device: Findings from a prospective cohort study in India. PLoS ONE 19(6): e0304120. <https://doi.org/10.1371/journal.pone.0304120>)*  Expulsion rate after vaginal PPIUD insertion was 2.92% at 6 weeks and 3.88% at 6 months of follow-up while intra-cesarean insertion had a 1.02% and 1.9% expulsion rate at 6 weeks and 6 months, respectively. Requests for PPIUD removal were higher in vaginal insertion cases. Discontinuation due to PPIUD removal and spontaneous expulsion was found to be 2.17% at 6 weeks follow-up and 4.2% at 6 months follow-up. However, overall 84.2% cases continued PPIUD as the method of contraception thus implying better acceptance by the society. Conclusion: PPIUD in the field of PPFP is a promising approach. Proper pre-insertion and postinsertion counseling regarding pain and bleeding can make clients tolerant to the side effects and improve the continuation rate. Counseling regarding spontaneous expulsion of PPIUCD is needed, which is more common after vaginal insertion, to prevent unwanted pregnancies. *(Shaidul Islam Borah, Alakananda Das, Ankita Bagchi: Outcome of PPIUCD insertion following vaginal delivery and intracaesarean: a comparative study. DOI -10.21276/obgyn.2024.10.2.22 ISSN Print – 2454-2334; ISSN Online – 2454-2342)*  The threads are not trimmed at insertion; therefore, there is a theoretical risk that they may protrude out of the vagina as the uterus involutes. In practice, this only occurred in 0.9% of cases in the largest study where 18 960 women were followed up. What is more common is that the thread becomes coiled up inside the uterus, particularly if the threads are not straightened after insertion during the cesarean delivery*. (Anita Makins, Sharon Cameron; Post pregnancy contraception; Best Practice & Research Clinical Obstetrics and Gynaecology; © 2020 Published by Elsevier Ltd)* | PPIUD counseling should provide comprehensive information on the device’s efficacy, benefits, potential side effects, and complications, addressing knowledge gaps and societal perceptions.  Counseling should include detailed discussions on common concerns such as menstrual irregularities, palpable threads, missing threads, and pain.  Since PPIUD threads are not trimmed at insertion, women should be advised that threads may protrude as the uterus contracts but that this is incredibly rare. Were it to occur, they should be instructed to gently push the threads back into the vagina and report to their provider.  In addition, HCPs should be made aware of the fact that because postpartum bleeding and discomfort are common postpartum and after insertion of the IUD, acceptance of the device may be higher immediately after childbirth than with interval insertion. |
|  | The method of choice should be administered at or immediately after birth (within 48 hours) and before discharge (WHO/FSRH/RCOG).  Maternity services with staff trained in postpartum insertion of intrauterine contraception and in the etonogestrel implant (ENG-IMP) should offer insertion of a LARC device to all medically eligible women before discharge from maternity services.  If women cannot be provided with their preferred method of contraception before discharge from maternity services, they should be offered effective bridging contraception and information about accessing local contraceptive services. *(Guidance on the provision of contraception by maternity*  *services after childbirth during the COVID-19 pandemic – FSRH, RCOG and RCMW 2021)*  A woman can receive a PPIUD at the time of cesarean delivery or within 48 hours of vaginal delivery. PPIUD insertion is not recommended between 48 hours and 6 weeks after delivery. *(FIGO - Technical Brief: Implementing postpartum intrauterine device (PPIUD) services in healthcare facilities)* | The chosen postpartum contraceptive method should be administered at or immediately after birth (within 48 hours) and before hospital discharge. If a PPIUD is not inserted within 48 hours of delivery, it should be delayed until 4–6 weeks postpartum.  If women are unable to receive their preferred contraceptive method before discharge from maternity services, they should be offered effective bridging contraception to ensure continuous protection. |
|  | PPIUD can be inserted in the uterus at cesarean delivery once the infant and the placenta have been removed and before the uterus is closed.  Refer to the FIGO PPIUD webpage with teaching videos.  The insertion of IUCD after cesarean delivery had an acceptable expulsion rate and there was no increase in the side effects rate compared to the IUD during natural vaginal delivery. Therefore, one can deduce that the post-cesarean IUD is as effective as the IUD immediate postpartum. *(Sajid DA, Khan SA, Sajid A, Sajid A. Comparison of Intrauterine Contraceptive Device Insertion After Normal Vaginal Delivery Versus Intra Cesarean. Pak Postgrad Med J 2024;35(2): 55-59, DOI: <https://doi.org/10.51642/ppmj.v35i02.696>)*  Post-placental insertion of an IUD during a cesarean delivery appears to be a more effective and convenient method of contraception compared to delayed insertion of an IUD. Our study has shown that post-placental IUD insertion is associated with lower rates of expulsion and thread visibility. *(Waleed M. Tawfik, Amal E. Ebrahiem, Mahmoud R. Fayed, Youssef A. Zaher: Post Placental Insertion of Different Types of Intrauterine Device During Cesarean Section versus Delayed Intrauterine Device Insertion in Sharkia Governorate. Article 7, Volume 41, Issue 4, July and August 2024, Page 66-75, DOI: 10.21608/bmfj.2023.233204.1886)*  For emergency obstetric care, many patients end up in cesarean deliveries and if not contraindicated, a PPIUD should be inserted. Of the total insertions, 61 308 (42%) were done during cesarean delivery and 85 010 (58%) after normal delivery. Total deliveries: 648 126, spontaneous vaginal deliveries: 345 540 (53.3%), lower segment cesarean sections: 294 364 (45.4%), assisted deliveries: 8222 (1.3%) (*Azra Ahsan and Aleya Ali; Acceptability, continuation and complication rate of postpartum insertion of intrauterine contraceptive device among Pakistani women; Journal of Pakistan Medical Association(Vol. 73, Issue 5); DOI: <https://doi.org/10.47391/JPMA.6804%20>; <https://jpma.org.pk/index.php/public_html/article/view/6804>*)  Latest available data (2010–2018) from 154 countries covering 94.5% of world live births shows that 21.1% of women gave birth by cesarean worldwide, with averages ranging from 5% in sub-Saharan Africa to 42.8% in Latin America and the Caribbean. Cesarean delivery has risen in all regions since 1990. *(Betran AP, Ye J, Moller A, et alTrends and projections of caesarean section rates: global and regional estimates BMJ Global Health 2021;6:e005671. doi:10.1136/bmjgh-2021-005671)* | With 21.1% of women worldwide giving birth by cesarean delivery — ranging from 5% in sub-Saharan Africa to 42.8% in Latin America and the Caribbean—the option to insert a PPIUD during cesarean delivery is an effective strategy. The PPIUD can be placed immediately after delivering the infant and placenta and before closing the uterus, with studies showing comparable effectiveness and expulsion rates to insertion after vaginal delivery. After insertion, care should be taken to not inadvertently include the IUD threads into closure of the incision.  Given the high rates of emergency cesarean deliveries, PPIUD insertion should also be integrated into EmONC services. If the woman has consented in advance and there are no contraindications such as prolonged rupture of membranes, evidence of chorioamnionitis or on going PPH, insertion of the PPIUC is safe to do. |
|  | PPIUD can also be inserted after vaginal delivery using the long Kelly forceps or the purpose-built device. It needs to be placed high in the fundus of the uterus to avoid expulsion. If it is correctly placed, there is evidence that expulsion rates should be no different to those having an interval insertion.  (Makins et al <https://obgyn.onlinelibrary.wiley.com/doi/full/10.1002/ijgo.12600>)  Providers should be trained on Mama-U models, using long-handled 33-cm curved Kelly forceps to ensure the IUD reaches the top of the fundus. Long-handled Kelly forceps are recommended, rather than 24-cm tissue or sponge forceps, which do not reach the fundus of the uterus leading to a higher chance of expulsion. *(FIGO - Technical Brief: Implementing postpartum intrauterine device (PPIUD) services in healthcare facilities)*  The long inserter PPIUD insertion is a safe and convenient method. It has better ease of insertion, high fundal placement, and good thread visibility and has reduced risk of infections compared to the conventional PPIUD insertion technique. (*Singh R, Yadav P, Sweta S, Singh S, Nigam A, Singh H. Clinical Outcome of Cu-T 375 PPIUCD by Novel Dedicated Inserter Technique. J Obstet Gynaecol India. 2021 Aug;71(4):430-436. doi: 10.1007/s13224-021-01445-6. Epub 2021 Mar 15. PMID: 34566304; PMCID: PMC8418579).*  The follow-up data of the current study are in contrast to an earlier study which reported an expulsion rate of 5.1% at 6 weeks and 7% at 6 months. *(Celen S, Möröy P, Sucak A, Aktulay A, Danişman N. Clinical outcomes of earlypostplacental insertion of intrauterine contraceptive devices. Contraception 2004; 69: 279-82. doi: 10.1016/j.contraception.2003.12.004.)* (*Azra Ahsan and Aleya Ali; Acceptability, continuation and complication rate of postpartum insertion of intrauterine contraceptive device among Pakistani women; Journal of Pakistan Medical Association(Vol. 73, Issue 5); DOI: <https://doi.org/10.47391/JPMA.6804%20>; <https://jpma.org.pk/index.php/public_html/article/view/6804>*)  In the present study, PPIUCD was found to be safe with an overall expulsion rate of 4.3%. It was marginally, though not significantly, higher in the immediate group. (*Dorairajan, Gowri; Ashok, Venkatesh M.; Veena, P.Effect of the timing of insertion of postpartum intrauterine contraceptive device (PPIUCD) copper T380A on expulsion rates. Indian Journal of Medical Research 157(4):p 322-329, April 2023. \| DOI: 10.4103/ijmr.IJMR_1485_19)*  Immediate PPIUD insertion (i.e. within 10 minutes after placental delivery in vaginal and cesarean births) should be offered routinely as a safe and effective option for postpartum contraception. *(ACOG Practice Bulletin, Number 186, November 2017)* | PPIUDs be inserted after vaginal delivery using either long Kelly forceps (33 cm) or a purpose-built device, rather than 24-cm tissue or sponge forceps, as the latter do not reach the uterine fundus, increasing the risk of expulsion. Proper placement high in the uterine cavity at the fundus is essential to minimize expulsion. When correctly positioned, evidence suggests that expulsion rates are comparable to those of interval insertions.  There is a better chance of thread visibility after insertion using the purpose-built device, but conversely they are also more likely to protrude from the vagina and so trimming is recommended at 2 weeks after insertion. |
|  | The PPIUD should not be inserted in the presence of infection (chorioamnionitis) or PROM (>18 hours) due to the risk of infection (RCOG).  PROM for more than 18 hours before delivery is listed as an exclusion criterion in most research studies about PPIUCDs. Because PROM increases the woman’s risk of postpartum uterine infection or puerperal sepsis, IUD insertion is not advised (Category 3). However, there is no strong or definitive evidence for this exclusion criterion, and thus it is open to review. *(Jhpiego Corporation, [www.jhpiego.org](http://www.jhpiego.org) – manual developed under Access FP program)* | PPIUDs should not be inserted in the presence of infection (chorioamnionitis) or PROM (>18 hours) due to the risk of infection. Additionally, if there is a history of prolonged labor, particularly when managed by traditional birth attendants with uncertain timings, PPIUD insertion should be avoided. |
|  | Complications rates with PPIUD are very low – infection x%, perforations not yet reported (add evidence) **[Author: Please check and update as necessary.]**  Interval IUC insertion (from 48 hours after childbirth) is associated with an increased risk of uterine perforation, particularly if the user is breastfeeding. However, the risk of uterine perforation from 28 days after childbirth remains small.  If more than 48 hours have passed since childbirth, insertion should be delayed until 28 days after childbirth (interval insertion). The risks of insertion from 48 hours until 28 days after childbirth generally outweigh the benefits (UKMEC3).  If IUD insertion has to be delayed due to infection, bridging contraception should be offered.  The rate of uterine perforation associated with IUD use is very low, with an overall risk of perforation in the general population of 1–2 in 1000.  Postpartum interval IUD insertion (from 48 hours after childbirth) is associated with an increased risk of uterine perforation, particularly if the user is breastfeeding.  *(FSRH Guidelines Intrauterine Contraception – Amended 2023)*  Pooling data across the six countries demonstrated that infection and perforation rates were minimal and expulsion rates were <3% (equal to interval IUDs). *(Makins A, Taghinejadi N, Sethi M, Machiyama K, Munganyizi P, Odongo E, Divakar H, Fatima P, Thapa*  *K, Perera G, Arulkumaran S. FIGO’s postpartum intrauterine device Initiative: Complication rates across*  *six countries. International Journal of Gynecology and Obstetrics. 2018. https://obgyn.onlinelibrary.*  *wiley.com/doi/full/10.1002/ijgo.12600 + FIGO - Technical Brief: Implementing postpartum intrauterine device (PPIUD) services in healthcare facilities)*  Not a single case of IUD-related infection was diagnosed in the current study, but IUDs were removed without any real evidence of infection. This was done due to mistaken perception of infection by the women and, more importantly, by the HCP concerned. Similar studies found no increase in the incidence of infection and removal of IUD. *(Welkovic S, Costa LO, Faúndes A, de Alencar Ximenes R, Costa CF. Post-partum bleeding and infection after post-placental IUD insertion. Contraception. 2001; 63:155-8. doi: 10.1016/s0010-7824(01)00180-9.)* + PPIUCD article.  In a study of 146 318 PPIUCD insertions, two cases of perforation were reported. (*Azra Ahsan and Aleya Ali; Acceptability, continuation and complication rate of postpartum insertion of intrauterine contraceptive device among Pakistani women; Journal of Pakistan Medical Association(Vol. 73, Issue 5); DOI: <https://doi.org/10.47391/JPMA.6804%20>; <https://jpma.org.pk/index.php/public_html/article/view/6804>*)  There are case reports of perforation after post-placental IUD insertion. Other studies, though with much smaller cohorts, did not report perforation or misplaced IUDs. *Çelen Ş, Sucak A, Yıldız Y, Danışman N. Immediate postplacental insertion of an intrauterine contraceptive device during caesarean section. Contraception. 2011; 84:240-3. doi: 10.1016/j.contraception.2011.01.006.)*  Eroglu et al. also reported no uterine perforation during immediate post-placental and early PPIUCD insertions. *(Eroğlu K, Akkuzu G, Vural G, Dilbaz B, Akın A, Taşkın L, et al. Comparison of efficacy and complications of IUD insertion in immediate postplacental/early postpartum period with interval*  *period: 1 year follow-up. Contraception. 2006; 74:376-81. doi: 10.1016/j.contraception.2006.07.003.)*  Trace the cervix in its entire circumference before inserting the IUD as the posterior fornix is deep and the IUD is perforated through the posterior vaginal fornix rather than the uterine wall.  Uterine perforation can be effectively prevented by hiring experienced HCPs and appropriate patient selection. *(Tabatabaei, F., Hosseini, S.T.N., Hakimi, P. et al. Risk factors of uterine perforation when using contraceptive intrauterine devices. BMC Women's Health 24, 538 (2024). <https://doi.org/10.1186/s12905-024-03298-3>)* | PPIUD insertions are associated with very low complication rates, with minimal risk of infection and very few cases of perforation reported. However, misperceptions among HCPs and patients can lead to unnecessary removal due to mistaken concerns of infection. In a large study of 146 318 PPIUD insertions, only two cases of uterine perforation were reported.  Proper training of providers and appropriate patient selection are essential to ensure safe and effective PPIUD insertion. To minimize the risk of perforation, it is recommended that HCPs carefully trace the entire cervix, before insertion to avoid accidental perforation, especially of the posterior vaginal fornix. |
|  | Women can self-check for the threads at 6 weeks and do not need to return for follow-up if all is well (FSRH).  With the exception of IUDs inserted within 48 hours of childbirth, routine post-insertion checks are not required. However, users should be advised to self-check their threads 4–6 weeks after insertion and then at regular intervals (e.g. monthly or after menses).  When the IUD is inserted within 48 hours of childbirth, an IUD check-up with a clinician 4–6 weeks after insertion is recommended as PPIUD is associated with an increased risk of expulsion and with long or non-visible threads. *(FSRH Guidelines Intra Uterine Contraception -2023)* | Routine post-insertion check-ups are not required for IUDs. When insertion occurs within 48 hours postpartum, a follow-up with a clinician 4–6 weeks after insertion is prudent due to the potential for longer or non-visible threads. |
|  | Lost threads can occur in up to 30% of cases but this rate should decrease as menstruation recommences (add EVIDENCE) – Missing Strings  The prevalence of non-visible threads may be as high as 18% (standard IUC insertion), 30% (IUC insertion within 48 hours of vaginal birth) and 50% (IUC insertion at the time of cesarean section). *(FSRH Guidelines Intrauterine Contraception – Amended 2023)*  At follow up, threads are not visible in 28 - 29% of cases, which includes insertion after vaginal and Cesarean births. There is also some anecdotal evidence that the threads often appear once menstruation resumes.( *Anita Makins, Sharon Cameron; Post pregnancy contraception; Best Practice & Research Clinical Obstetrics and Gynaecology; © 2020 Published by Elsevier Ltd)*  Missing threads were detected more in cesarean group (30%) than vaginal group (19%). PPIUCD is very effective, safe and reversible contraceptive method which provides contraceptive effect soon after birth. The PPIUCD is a long-acting reversible contraceptive method that is suitable for use in all women in postpartumperiod. Missing strings after PPIUCD insertion is a common problem encountered during follow up examination and better management has to be provided. *(Dalal, R., Chavda, N. and Desai, A.N., Prospective Study for Evaluation of PPIUCD Insertion as a Method of Contraception. Dr. Devendra R. Patel, p.31.)*  Hysteroscopy is a good modality for the removal of PPIUD with lost strings. *(Perveen, A. ., Zakaria, R. E., & Begum, H. . (2022). Tales of Hysteroscopic Evaluation of PPIUD with Missing String in a Tertiary Care Government Hospital. Journal of Shaheed Suhrawardy Medical College, 13(1), 63–67. <https://doi.org/10.3329/jssmc.v13i1.60934>)*  Threads may not be visible in up to 50% of individuals following intra-cesarean PPIUC insertion and up to 30% of individuals following postpartum vaginal insertion. This reflects the differences in insertion technique and anatomical changes occurring during the postpartum period. In some cases, the threads may descend into the vagina over time as the uterus returns to its non-pregnant state and menstruation resumes. Even in the absence of visible threads on speculum examination at follow-up, most devices will be correctly positioned in the uterus, but IUC expulsion needs to be excluded. *(FSRH Guidelines Intra Uterine Contraception -2023)*  If the threads are not palpable the lady should be advised to use alternative contraception until she can go for a check up. If on examination no threads are evident, an ultrasound should be performed to exclude expulsion. If the device in in situ the threads can be retrieved with a thread retriever or cyto brush once the lady decides she wants the coil removed. (FIGO PPIUD initiative). – Cytobrush (RCOG/FSRH ref). It is advised that thread retrieval is performed by senior clinicians only as 2 cases of perforation over 146 000 insertions were reported following the use of metal thread retrievers and artery forceps for retrieval of lost threads (personal communication).  Women who have undergone insertion of intrauterine contraception after childbirth may require follow-up for pain, bleeding, or expulsion, or if threads are very long or cannot be felt. *(Guidance on the provision of contraception by maternity services after childbirth during the COVID-19 pandemic – FSRH, RCOG and RCMW 2021)*  Missing CuT thread is commonest with postpartum IUCD. TVS should be first investigation in missing CuT thread. X-ray abdomen is needed only if CuT is not visualized on TVS. Hysteroscopy can be used in patients where CuT retrieval with artery forceps fails. Laparoscopy and laparotomy may be required in migrated CuT cases. *(Stings of the missing string: missing Copper T thread; Ketaki K. Junnare, Saloni Savaskar, G. S. Shekhawat DOI: http://dx.doi.org/10.18203/2320-1770.ijrcog20196004)* | Inform patients that missing strings or lost threads can occur in up to 30% of postpartum IUD cases, especially among those who had a cesarean delivery. This rate often decreases once menstruation resumes.  In order to minimise lost threads following CS insertion it is advised that once the device is placed at the fundus of the uterus, that the threads are straightened towards the internal os.  Missing threads are generally not a cause for concern; patients should be reassured, and further intervention is only needed if IUD removal is required. For management, transvaginal sonography (TVS) is advised as the first-line investigation. If the device is not visible on TVS, an abdominal X-ray may be necessary. In instances where retrieval with artery forceps/ thread retriever/ Cytobrush is unsuccessful, hysteroscopy can be considered. For cases involving migrated devices, laparoscopy or laparotomy may be required. |
|  | Task sharing is a valuable tool to increase access to PPIUD services  MWs equally competent:  MWs, when trained and empowered, were equally skilled in providing safe PPIUD services. As such, task sharing or shifting with MWs is another effective strategy to improve uptake of PPFP services.  (*Azra Ahsan and Aleya Ali; Acceptability, continuation and complication rate of postpartum insertion of intrauterine contraceptive device among Pakistani women; Journal of Pakistan Medical Association(Vol. 73, Issue 5);*  *DOI: <https://doi.org/10.47391/JPMA.6804%20>; <https://jpma.org.pk/index.php/public_html/article/view/6804>*)  Once trained, PPIUDs can be inserted by mid-level providers through task sharing. Task sharing is strongly recommended, especially in facilities where women will only come into contact with MWs during delivery. During the FIGO PPIUD Initiative, introducing task sharing in India increased PPIUD uptake from <1% to 37% and in Tanzania more than 58% of insertions were conducted safely by MWs. *(FIGO - Technical Brief: Implementing postpartum*  *intrauterine device (PPIUD) services in healthcare facilities)*  *(Bhadra B, Burman S, Purandare C, Divakar H, Sequeira T, Bhardwaj A. The impact of using nurses to perform postpartum intrauterine device insertions in Kalyani Hospital, India. 2018. <https://obgyn.onlinelibrary.wiley.com/doi/full/10.1002/ijgo.12602>)*  *Muganyizi PS, Kimario G, Ponsian P, Howard K, Sethi M, Makins A. Clinical outcomes of postpartum*  *intrauterine devices inserted by midwives in Tanzania. International Journal of Gynecology and Obstetrics.*  *2018. <https://obgyn.onlinelibrary.wiley.com/doi/10.1002/ijgo.12603>)* | Task sharing is strongly recommended as an effective approach to expand access to PPIUD services. With proper training and empowerment, mid-level providers are equally skilled in delivering safe PPIUD services. This is particularly beneficial in facilities where women primarily interact with MWs during delivery. |
|  | Continuous supply chain and availability of contraceptives round the clock in the labor room.  The system for continuous supply of contraceptives with a working supply chain should be in place, and contraceptives should be available in labor rooms and operating theaters around the clock. Currently, HCPs are not aware as to how to get a sustainable supply of contraceptive commodities for their HFs. This should be addressed as a priority. *(Azra Ahsan and Aleya Ali; Acceptability, continuation and complication rate of postpartum insertion of intrauterine contraceptive device among Pakistani women; Journal of Pakistan Medical Association(Vol. 73, Issue 5); DOI: <https://doi.org/10.47391/JPMA.6804%20>; <https://jpma.org.pk/index.php/public_html/article/view/6804>*  History tells us that in previous large infectious outbreaks, such as the recent Ebola epidemic in DRC, Sierra Leone, and Liberia, contraception and routine maternal health care dropped dramatically. The well-described “three delays” in obstetric care were exacerbated, and the reluctance to come for antenatal, postnatal, and FP consultations was clearly seen. A second major area of concern is the impending stock‐outs of contraceptive methods, which are about to ensue. UNFPA estimates that during the next 6 months, 46 countries that usually receive supplies from them will experience stock‐outs of one or more modern methods, including: implants; depot medroxyprogesterone acetate (DMPA) intramuscular (IM) and subcutaneous (SC); copper IUDs; oral contraceptive pills (combined and progestin only); and condoms. Authors at the Guttmacher Institute have estimated that if there were a 10% decline over the course of 1 year in the use of contraception due to stock‐outs, unavailable providers, or closed clinics, an additional 48 558 000 women would have an unmet need for contraception worldwide, resulting in 15 401 000 additional unintended pregnancies, 1 745 000 additional women experiencing major obstetric complications without care, and 3 325 000 additional women resorting to unsafe abortions. This would set the world back in terms of what had already been achieved by the Millennium Development Goals and make the challenge of meeting the Sustainable Development Goals even more difficult. (*Makins A, Arulkumaran S; FIGO Contraception and Family Planning Committee. The negative impact of COVID-19 on contraception and sexual and reproductive health: Could immediate postpartum LARCs be the solution? Int J Gynaecol Obstet. 2020 Aug;150(2):141-143. doi: 10.1002/ijgo.13237. Epub 2020 Jun 25. PMID: 32449192; PMCID: PMC9087606.*  *<https://pmc.ncbi.nlm.nih.gov/articles/PMC9087606/>)*  Recommended criteria for facility selection: There is already an IUD supply chain,  Supportive local/national government policy.  *(FIGO - Technical Brief: Implementing postpartum intrauterine device (PPIUD) services in healthcare facilities)* | To ensure the continuous availability of contraceptives, a robust supply chain system should be established, guaranteeing that contraceptives are accessible in labor rooms and operation theatres 24/7. Currently, HCPs lack clarity on how to secure a sustainable supply of contraceptive commodities for their facilities, and addressing this gap should be prioritized. |
|  | Pre-service.  Both pre-service and on-the-job training are essential to maintaining provision, as is ensuring adequate  general staffing levels. *(FIGO - Technical Brief: Implementing postpartum intrauterine*  *device (PPIUD) services in healthcare facilities)*  HCP (nursing and medical) pre-service curriculums should be updated to include PPFP and  trainees should receive in-service training on PPFP. *(Kabra R, Pradhan P, Hyder MKA et al. Gaps and*  *evidences on programming postpartum family planning services in Nepal. Gates Open Res 2022,*  *6:84 (<https://doi.org/10.12688/gatesopenres.13606.1>)*  Integrate concepts of PPFP within pre-service education and ensure that PPFP and HTSP are well-covered  in teaching curricula, practical training, and examinations *(WHO - Programming strategies for postpartum*  *family planning 2013)* | To sustain the provision of PPFP, including PPIUD, integrate both pre-service and on-the-job training for health providers. Nursing and medical curricula should be updated to incorporate PPFP concepts into pre-service education, practical training, and examinations. |

Abbreviations: ANC, antenatal care; EmONC, Emergency Obstetric and Newborn Care; FCHV, female community health volunteer; FP, family planning; HCP, healthcare provider; HF, healthcare facility; IUD, intrauterine device; LAM, lactation amenorrhea method; LARC, long-active reversible contraceptive; LMIC, low- and middle-income country; LNG-IUS, levonorgestrel intrauterine system; MW, midwife; PPFP, postpartum family planning; PPIUD, postpartum intrauterine device; PROM, prolonged rupture of membranes; SBA, skilled birth attendant; IUS, intrauterine system.
